# Advances in Functional Rubber and Elastomer Composites, 3rd Edition

**DOI:** 10.3390/polym18040486

**Published:** 2026-02-14

**Authors:** Md Najib Alam, Vineet Kumar

**Affiliations:** School of Mechanical Engineering, Yeungnam University, 280, Daehak-ro, Gyeongsan 38541, Republic of Korea; mdnajib.alam3@gmail.com

## 1. Introduction

Rubber and elastomer matrices are widely used in advanced engineering and technological applications due to their robust mechanical flexibility and favorable electrical properties [1,2,3,4,5]. Tailoring the functionalities of both the rubber matrix and the incorporated fillers can lead to synergistic enhancements in composite performance, producing properties that surpass those of traditional materials [6,7,8,9,10,11]. For instance, carbon materials exhibit excellent electrical characteristics but inherently lack stretchability. When combined with suitable elastomer matrices, such composites are mechanically compliant while retaining high piezoresistive sensitivity [12,13,14,15,16].

Dielectric elastomers represent an important class of materials that function as electromechanical transducers, with considerable potential in soft robotics and actuator technologies [17,18,19,20,21,22,23]. More recently, rubber-based materials have also been explored as stretchable triboelectric systems for energy harvesting and self-sensing electronic applications [24,25,26,27,28,29,30,31]. The enhanced mechanical robustness and electrical performance of these composites are largely attributed to filler functionalization, which improves filler–rubber interfacial interactions and promotes uniform filler dispersion within the elastomer matrix [32].

In our previously published editorials, we briefly discussed recent advances in rubber and elastomer composites exhibiting mechanical, magneto-mechanical, electrical, thermal, dielectric, energy-harvesting, sensing, and other multifunctional properties relevant to advanced applications [33,34]. This concluding editorial further builds upon those discussions by critically examining recent developments, with particular emphasis on the roles of rubber matrices and functional fillers in governing the structure–property relationships of these composite systems.

## 2. Overview of Published Articles

Nakyp et al. [35] studied carbon-enriched shungite concentrates derived from rare-metal mining waste and their effects on oil- and fuel-resistant carbon-black-filled rubbers for pressure hoses. Shungite fillers (5–15 phr), produced by flotation and acid activation, partially replaced the carbon black in nitrile butadiene rubber and butadiene–α-methylstyrene rubber (NBR/BR) blends. Shungite increased scorch and optimum cure times and generally reduced Mooney viscosity, while acid-activated shungite above 5 phr increased viscosity. Acid-activated shungite improved tensile strength, thermo-oxidative stability, and oil resistance, whereas raw shungite above 5 phr degraded tensile performance. Marín-Genescà et al. [36] studied the structural and thermal–dielectric behavior of styrene–butadiene rubber (SBR) and SBR blends with devulcanized ethylene–propylene–diene monomer (SBR/EPDMd) composites containing SiO_2_ with varying EPDMd contents. Distinct DC (direct current) and AC (alternative current) conductive regimes were observed, reflecting different dielectric responses at low and high frequencies. Polarization-related relaxation peaks appeared in the imaginary modulus spectra. Although SiO_2_ had little effect on electrical conductivity, it significantly modified the permittivity and electrical modulus, indicating changes in the energy storage behavior. Compared with silica-free systems, SiO_2_-containing composites exhibited reduced the polarization effects and dielectric losses, resulting in materials that are more electrically insulating and suitable for insulation applications. Kayacı et al. [37] investigated the hyperelastic and viscoelastic behavior of carbon-black-filled natural rubber (50 and 60 Shore A) using cyclic shear/compression and stress relaxation tests. The Arruda–Boyce model accurately described equilibrium behavior, while the Bergström–Boyce model captured transient viscoelasticity without using Prony series. Hysteresis analysis showed 7–26% energy dissipation, depending on hardness and strain rate, with relaxation rates (10^−6^–10^−7^ s^−1^) inversely related to hysteresis. Finite element simulations agreed well with the experimental results, with deviations below 3.5%, demonstrating the model’s reliability for long-term viscoelastic predictions and durable rubber component design. Khamjapo et al. [38] investigated the mechanical properties and biodegradability of sulfur-vulcanized polyhydroxyalkanoates (PHAs) with unsaturated side chains. Poly(3-hydroxybutyrate-co-3-hydroxy-5-hexenoate) with varying unsaturation levels (3–47 mol%) was biosynthesized and vulcanized using different sulfur contents. Crosslink formation was confirmed using chloroform insolubility and Raman spectroscopy. Vulcanization markedly improved tensile strength and elongation at break, reaching 6.3 MPa and 813% at 5 phr sulfur, respectively. Although biodegradability decreased with increasing crosslink density, vulcanized PHAs retained higher degradation potential than conventional vulcanized rubber, demonstrating rubber-like elasticity with partial biodegradability. Zhang et al. [39] compared continuous and discrete spectral methods for determining viscoelastic response functions in asphalt mixtures. Using complex modulus testing and generalized sigmoidal master curves combined with approximated Kramers–Kronig relationships, both the creep compliance and relaxation modulus were evaluated. The discrete spectra derived from Prony series and continuous spectra obtained through integral transformations showed strong agreement within conventional time scales (10^−7^–10^5^ s). Deviations emerged only at extreme time limits, where continuous spectra asymptotically approached the upper and lower plateaus. Crumb-rubber-modified asphalt exhibited superior low-temperature cracking resistance, whereas SBS-modified asphalt showed stronger high-temperature deformation resistance. The study highlights the strengths and limitations of spectral methods for accurate viscoelastic characterization. Bakošová et al. [40] explored the effect of single-walled carbon nanotubes (CNTs) on the mechanical performance and thermal aging resistance of natural rubber composites in sealing applications. CNT loadings of 1–4 phr led to increases in tensile and tear strength of up to 11.73% and 14.64%, respectively, while reducing aging-induced strength degradation by 5%. CNT incorporation increased the hardness and complex modulus but reduced the elongation at break and rebound resilience. The residual deformation decreased in tensile and compression set tests, and AFM analysis confirmed enhanced surface stability after thermal aging. Huang et al. [41] conducted a comprehensive review of polyurethane (PU)-foam-based flexible strain sensors, emphasizing the role of conductive fillers in enabling piezoresistive, piezoelectric, and capacitive sensing mechanisms. The review covers material composition, fabrication techniques, sensing performance, and applications in motion detection, health monitoring, and industrial sensing, while addressing current challenges and future prospects. Finally, Wang et al. [42] reviewed the recent advances in rubber fatigue research, highlighting the mechanisms of crack initiation and propagation under cyclic loading. They synthesized studies from the past decade, identifying emerging trends, experimental techniques, and research gaps, particularly in advanced rubber composites such as magnetorheological elastomers, providing valuable guidance for fatigue life prediction and engineering applications.

## 3. Summary and Future Outlook

Recent studies have highlighted substantial advances in rubber and elastomer composite research across diverse applications. Carbon-enriched shungite concentrates can be used to partially replace the carbon black in oil- and fuel-resistant rubbers, with acid-activated shungite significantly increasing tensile strength, thermo-oxidative stability, and oil resistance. Incorporating SiO_2_ into SBR/EPDMd composites strongly influences dielectric behavior by reducing polarization’s effects and dielectric losses, providing materials suitable for insulating applications. The hyperelastic and viscoelastic behaviors of carbon-black-filled natural rubber have been accurately described using advanced constitutive models, enabling the reliable long-term prediction of their performance. The sulfur vulcanization of polyhydroxyalkanoates markedly enhances their tensile strength and elasticity while retaining partial biodegradability, offering sustainable rubber-like alternatives. Comparative spectral analyses have clarified the strengths and limitations of continuous and discrete methods for the viscoelastic characterization of rubber-modified asphalt. The addition of low loadings of carbon nanotubes increases the mechanical strength and thermal aging resistance of natural rubber composites. Recent reviews have further summarized the progress in flexible polyurethane-foam-based strain sensors and in understanding rubber fatigue mechanisms, providing guidance for future composite design and engineering applications.

Various improvements in materials and their properties have been achieved; therefore, future efforts should focus on their application in advanced engineering and technological fields.
